# Self-spoliation and forms of resistance in total institutions: an exploration of time and space in an Albanian communist regime internment camp

**DOI:** 10.3389/fsoc.2025.1393612

**Published:** 2025-08-13

**Authors:** Federica Floridi, Silvia Cataldi, Marino Bonaiuto, Alessandra Talamo

**Affiliations:** Department of Psychology of Developmental and Socialisation Processes, Sapienza University of Rome, Rome, Italy

**Keywords:** extreme contexts, temporisation mechanisms, place attachment, self-spoliation, identity resistance, total institutions

## Abstract

The paper explores the daily life within Albanian internment camps during Enver Hoxha's prolonged communist regime, covering the period from 1944 to 1985. Extensive in-depth interviews with former internees underpin the research, which investigates the multifaceted strategies employed by these captives to resist the totalitarian obliteration of time and space, inherent in total institutions. These camps were not merely sites of physical isolation but were ideologically conceived as instruments of political repression, mirroring the Soviet Gulag system. The importance of community relationships for the survival of ex-internees is highlighted, featuring a deep network of mutual aid and long-lasting friendships. This serves as a sharp contrast to the oppressive context. The study reveals a paradoxical dimension to the ex-internees' experience: a strong attachment and reverence toward the locations that brought them great suffering, almost deeming them sacred. In these spaces, solidarity became a form of collective psychological resistance, allowing individuals to reconstruct emotional integrity and assert autonomy despite the brutal regime. This connection to sites of suffering establishes a fundamental foundation of personal and social identity, showcasing the incredible strength of humanity even in the harshest circumstances. It also elucidates the opposing forces of subjugation and resistance within the camp system, revealing the transformative strength of communal ties and the singular bond with these sites of affliction. In exploring these themes, it delves into the complex interplay between individual identity, collective solidarity, and the profound impact of extreme contexts. These findings challenge traditional views on total institutions, emphasizing the dynamic and active role of memory in survivor narratives, where personal and collective histories are reconstructed within the framework of trauma and emotional resilience.

## Introduction

The communist regime dictatorship from 1944 to 1985 under Enver Hoxha (1904–85) was characterized by severe repression of dissent through thousands of killings, imprisonments, and deportations to internment camps even for those who were merely suspected of differing opinions.

The Institute for the Study of the Crimes and Consequences of Communism in Albania (ISKK^1^) approximates that there were 30,000 to 34,000 political prisoners in Albania, comprising 26,700 men and over 7,000 women. According to the same source, 5,577 men and 450 women received death sentences and were executed. The bodies of those executed or who perished in prison, during forced labor, or due to illness were never returned to their families. In total, the number of people killed or imprisoned during the 41-year dictatorship amounted to over one-thirtieth of the Albanian population of that period. The Municipality of Lushnjë, the site of 14 internment camps, was one of the largest areas of political repression during the communist regime in Albania.

These camps were not merely sites of physical isolation; rather, they were ideologically conceived as instruments of political repression. Adhering to the regime's ideological underpinnings, the Albanian administration drew inspiration from the Soviet Gulag system, utilizing internment as a means not solely to punish those who voiced opposition but also to indoctrinate the internees. The function of these camps was to act as sites for the re-education of the populace, with the aim of creating an individual who embodied the qualities of the new communist person. However, it should be noted that this objective was largely a rhetorical device. In practice, the role of these institutions was to facilitate the dissolution of individual identity through the imposition of forced labor, surveillance, and the implementation of dehumanizing routines (Chodownik, 2018; Abram and Bassi, 2010; ISKK, 2023). Following Hoxha's death and the protests that erupted in the country from 1989 onwards, the first democratic elections in 1991 marked the end of the communist regime and Albania's reintegration into the international community.

Thirty years after the end of one of the most dramatic periods in Albanian history, the processing of the suffering experienced is still in its infancy. In this process, undoubtedly influenced by the memory of the horrors of the dictatorship period, an essential part is represented by the testimonies of those who experienced the suffering personally, particularly in the internment camps. Within the camps, the spatial and temporal organization was deliberately structured to reflect the ideological goals of totalitarian control. The daily routine was fragmented into repetitive and exhausting tasks, regulated by strict schedules, and supervised by guards and informants. Spatially, the camps were designed to isolate and degrade overcrowded barracks, lack of sanitation, and harsh climatic exposure reinforced the sense of abandonment and social isolation. This spatial-temporal architecture was essential in enforcing obedience and discouraging resistance (Paweczynska and Pawełczyńska, 1979; ISKK, 2023).

In recent years, the contemporary relevance of this issue has been amplified by the opening of the communist regime's archives, which has allowed many Albanian citizens to uncover documents concerning themselves and their families. These revelations have often brought to light betrayals by friends or relatives, causing renewed trauma and profound emotional distress, as the secrets of Hoxha's henchmen continue to poison Albanian society (France 24, 2022).

By shedding light on modes of resilience based on group cohesion and solidarity between those who faced the same situations of dehumanization, deprivation and exclusion, these testimonies attribute fundamental importance to the dimensions of time and space. These dimensions are at the basis of processes and dynamics that have enabled the re-elaboration, in positive experience, of the horrors experienced. These are, therefore, mental processes that in many cases have allowed not only survival, but also the creation of strong social bonds that have remained imprinted in individual and collective memory. The “positive” memories of the period lived in the camps and the attachment to the places of suffering thus become fundamental elements in the process of constructing individual and social identity.

In response to these still-open wounds, various artistic and cultural initiatives have emerged to support a collective reworking of memory. For instance, the installation “Even Walls Have Ears”, simultaneously projected in six Albanian cities, used the testimonies of former detainees to commemorate victims and promote European values. Furthermore, the transformation of Enver Hoxha's former villa in Tirana into an art center that hosts international creatives represents a potent symbol of reconciliation with the past. As reported by AP News, “the former communist dictator's house now hosts what he most despised: free thinkers”, underlining the key role of art in collective healing processes (Semini, 2021; UNDP, 2021).

Studies on concentration camps have predominantly focused on the structural and institutional aspects, overlooking what could be referred to as the psycho-social dimension of the internees' lives (Pingel, 2009). While specific studies on Albanian internment camps remain relatively limited, a comparative framework with Soviet Gulags is crucial. Both systems shared fundamental characteristics: ideological indoctrination, forced labor as punishment and “reeducation”, and an architecture of surveillance and confinement. However, scholars suggest that the Albanian system was even more isolated and ideologically rigid, given Hoxha's radical break with both the Soviet Union and China in the 1960s and 1970s, which led to an autarkic and highly isolationist version of communism (Abram and Bassi, 2010; Fischer, 2010; Kadare, 2006).

However, the topic addressed in this article—specifically, the social aspect of everyday life in internment camps during the Albanian communist regime—cannot be dissociated from the camp's structure and two fundamental concepts: space and time. Paweczynska and Pawełczyńska (1979) explored the relationship between the environment and individuals in their work on the Auschwitz concentration camp, emphasizing the significance of space (both physical and social) and time as central to their analysis. Consequently, some authors have examined life in Nazi concentration camps through the lens of the physical-spatial element, recognizing its importance in understanding the inmates' attitudes and assessing their chances of survival, within environments characterized by the principles of confinement and complete control over space and time (Messina, 2017, p. 132).

Building on these assumptions, this article explores the complex two-way relationship between self and social context (Harrison, 2009, p. xxiii) within the places and rhythms dictated by the total institution of an Albanian internment camp under the communist regime. Through the hermeneutic analysis of interviews conducted with survivors in the Savër camp in Lushnjë, the mechanisms of spoliation and identity resistance narrated by the internees will be examined. In this context, to fully understand the term self-spoliation, it is crucial to consider Erving Goffman's concept of “spoliation”. With self-spoliation, we specifically refer to the spoliation of the self, that is, the individual. Goffman (1961) describes spoliation as a ritualized process of emptying personal identity, in which individuals gradually and systematically lose not only their material possessions but also their emotional connections, social roles, and autonomy. It is within this framework that internees undergo an imposed spoliation of self, only to strive for forms of identity preservation and resilience through shared practices and communal bonds.

The article, therefore, focuses on the daily life of the internees, paying particular attention to two (de)structuring dimensions of experience: time and space. The temporal mechanisms typical of total institutions in the scanning of life will be analyzed, and forms of resistance to the colonization of everyday life will also be identified. Concerning the spatial dimension, in addition to describing the structure of the camp and the degradation of the barracks, the bond that was created between internees and place and the role that this attachment played in the construction of personal and social identity will be highlighted.

The analysis also highlights how memory becomes a dynamic and affective reconstruction of the past, shaped by present-day identities and the emotional resonance of testimony, rather than functioning as a static recollection. Furthermore, the article explores how practices of solidarity and emotional closeness enabled internees to combat the isolation imposed by the regime, serving as tools of resistance and resilience. These practices frequently manifested as micro-gestures of care, shared routines and collective efforts to preserve dignity and agency in the face of systemic dehumanization. Finally, by linking the lived experience of the past to its contemporary interpretation, the article sheds light on how the trauma of internment continues to reverberate today, particularly through personal and familial memories, the reclamation of formerly marginalized spaces, and recent cultural initiatives aimed at healing and achieving public recognition.

After a theoretical introduction, the methodology adopted will be presented. The main findings will then be illustrated in three sections: confinement and living conditions in the camp; everyday life of the internees; identity and relationship with the place.

## Time and space in total institutions

The structuring of time and space undergoes severe alteration within institutions, such as the internment camps established under the communist regime in Albania. Erving Goffman's (1961) influential work, Asylums, offers key insights into the exclusion and violence mechanisms that are characteristic of the world of the interned. Through the well-known ethnography conducted in a Washington DC psychiatric hospital in the United States, the author introduces the concept of total institution intended as a place of residence and work where like-situated individuals, cut off from the wider society for a significant duration, together lead an enclosed, formally administered round of life (Goffman, 1961, p. xiii). The main characteristic of such institutions is their ability to exert control over every significant part of the inmate's life, including space and time (Smith, 2006, p. 103).

Spatial confinement goes beyond physical restraint; it encompasses symbolic and affective segregation. Initial confinement is evident in physical barriers like walls, barbed wire, and barriers that isolate inmates from the civilized world, preventing interaction with the external environment. Additionally, spatial manipulation and constriction occur through centralized authority dictating the performance of various activities in designated places, strict control of communication, regulation of internal movements, prohibition of personalization in living spaces, and severe restrictions on the use of personal items, favoring those provided or permitted by the institution.

These spatial constraints have deep psychosocial implications. As Crewe (2011) points out, the management of space in prison is directly linked to the experience of emotional regulation, where the denial of privacy and autonomy contributes to feelings of humiliation and helplessness. The inability to inhabit a space as one's own undermines not only bodily agency but also the symbolic construction of identity, affecting the “depth” of social separation, the “weight” of psychological oppression, and the “tightness” of invasive control (Crewe, 2011, p. 513–522).

Time also plays an essential role in the regimentation of total institutions. From the admission procedures onward, inmates experience a loss of control over daily life and an annihilation of time freedom. The institutions enforce rigid schedules, strict surveillance, and numerous obligations from early morning till late evening. Daily activities are carried out in large groups under close supervision, guided by a system of strict rules that enforce repetitive events, standardized tasks, and uniform behavior. In this manner, the organization seizes control over daily routines, incorporating the interests and energies of those within its confines.

The forced temporality, often described by inmates as “doing dead time” (Jewkes, 2005, p. 372), contributes to a temporal trauma that fragments personal continuity. The alienation from one's own biographical time hinders future orientation and anchors the subject in a suspended present devoid of meaning and agency. As Fiddler (2011) notes, the institutionalization of chronemics acts not only as a mechanism of control but as an architecture of emotional disempowerment.

In such carceral settings, the material and symbolic configuration of the environment profoundly reshapes subjective experience. As Moran (2013) points out, the spatial organization and the mechanisms of control produce a liminal condition that simultaneously disrupts social relations and opens up ambiguous possibilities for contact and resistance. These spaces are deliberately structured to displace and isolate, inscribing a sense of uprootedness into bodies and emotions. Haney (2018), in his analysis of the effects of extreme isolation, illustrates how the loss of spatial, temporal, and relational reference points leads to psycho-cognitive disturbances and a deep erosion of single identity. Medlicott (2001) underscores that such geographical environments are far from neutral: sensory deprivation, enforced inactivity, and the compression of space-time experience generate emotional tension, frustration, and a disorientation of the self. Under such conditions, time no longer flows according to subjective rhythms but is instead imposed as an institutional form of discipline, contributing to the erosion of personal continuity and the fragmentation of subjectivity.

Space and time, therefore, represent the levers through which total institutions manipulate and mortify the self of the inmate, penetrating each person's being with its expressions and transformations. Upon their entry, inmates endure a series of humiliations, degradations, and profanations that force the abandonment of their identity connected to civilized life. Simultaneously, their perception of self and relationships with others undergo radical transformations.

These transformations are not only social but profoundly affective and embodied. Trauma theorists such as Herman (1992) have shown how captivity can produce dissociative states, emotional numbing, and identity diffusion. In carceral contexts, these psychic responses are tightly entangled with the spatial and temporal logics of incarceration, where the loss of self is not just symbolic but lived in the rhythms and architectures of confinement.

However, this transformation is not solely a process of passive leveling. Upon closer examination, Goffman (1961) considers that some identity mechanisms are also activated in total institutions, with subjects as protagonists. Although these dynamics are only briefly described by Goffman and re-evaluated only by more recent literature (Shreeya, 2018; Elliott, 2012; Scott, 2011; Smith, 2006), they are essential because—acting precisely on spatial and temporal dimensions—they highlight an ever-living reveal an inherent contradiction between the organizational pressure for self-abnegation and the agentic processes of identity construction.

The tension between the institution's temporal-spatial ordering and the subject's affective and mnemonic life becomes the terrain where identity is reasserted through micro-practices of resistance. As Moran et al. (2012) emphasize, even within tightly controlled carceral architectures, individuals find ways to reclaim emotional agency and biographical coherence by subverting or re-signifying the temporal markers of institutional life.

One protective mechanism employed by internees involves reactions such as irony, contempt, and mockery to cope with mortifying situations, establishing a distance between the self and the spoliation to which they are subjected (Smith, 2006, p. 75). While these reactions may attract further assaults and repressions, they are crucial for preserving the sense of being from depersonalization caused by internal rules and regulations and for separating agentic action from the conflation of space-time dimensions (Goffman, 1961).

In addition to embodying institutional expectations, residents often resist these definitions, striving for the recognition of their dignity a form of “resistance to oppression” (Dal Lago, 2001, p. 6) or the “countervailing self” (Smith, 2006, p. 103). Goffman describes this as individuals creating a space between themselves and how others tend to identify them (Goffman, 1961, p. 132). Acts of resistance involve adapting to the situation through underground strategies, cultivating personal interests, and fostering connections with people, places, and circumstances.

Such acts often express themselves in deeply affective forms—letters, secret rituals, moments of silence or laughter—through which inmates rebuild alternative temporalities and reinhabit spaces with memory and emotion. These emotional geographies of resistance contest the institutional claim to total control, as argued by (Crewe and Bennett 2012), offering fragments of personal continuity and re-identification.

These adjustments, often seen in small acts of personalizing space and time, constitute underlife-practices that escape regimentation within total institutions. Goffman (1961) argues that these activities are not just defense mechanisms but essential signals of self-construction (Goffman, 1961, p. 319). Despite their minimal nature, these activities enable individuals to resist, or challenge assigned identities imposed by institutions, asserting their sense of self.

It is precisely through this embodied and emotional reworking of time and space that we witness the enduring struggle for identity under confinement—where the carceral condition is not merely endured, but also interpreted, remembered, and emotionally negotiated.

The following paragraphs will delve into the antinomian processes of self-spoliation and identity resistance narrated by survivors of the Albanian communist regime's internment camps, building upon the importance of these mechanisms in total institutions.

## Method

This study is based on an analysis of in-depth interviews. It gathered testimonies from survivors of Albanian internment camps under Enver Hoxha's totalitarian regime, aiming to connect history and memory to the contemporary construction of European identity and citizenship.

Twenty-seven in-depth interviews were conducted with twenty-one men and six women between the ages of 51 and 83. All the interviewees lived in the camp in Lushnjë, although some of them were interned, for longer or shorter periods, in other camps in the country.

The interviews were mainly conducted face-to-face (in different cities in Albania: Lushnjë, Durres, Tirana, Vlora), except in cases where online interviews were conducted through the Zoom platform as some interviewees no longer live in Albania but reside in Italy, the United States and Canada. Today, these individuals live in diverse geographical and social contexts, leading lives that appear outwardly normal and integrated. Yet, they continue to carry a deep and enduring wound rooted in their internment experience. This painful memory played a crucial role in motivating their willingness to participate in the study. For many, the act of telling their story represents both a contribution to preventing such atrocities from happening again in the future and a personal opportunity to process and give significance to their trauma.

These narratives are embedded within a cultural context marked by decades of enforced silence, intergenerational trauma, and a still-contested public memory of the communist past. The act of remembering and speaking out today is shaped by ongoing societal tensions around recognition, reconciliation, and the role of memory in national identity.

In light of the sensitive nature of the topics addressed, all interviews were conducted in the presence of a licensed clinical psychologist, who was available to provide professional support in the event of emotional distress or reactivation of traumatic experiences during the interviews. The psychologist's presence was essential in safeguarding the participants' psychological well-being and in creating an atmosphere of respect, emotional safety, and containment throughout the research process.

Participants were contacted through a mediator affiliated with a partner NGO involved in the project. This organization has long collaborated with communities of former internees and annually promotes a public commemoration of the closing of the internment camps. These pre-existing relationships were instrumental in establishing a climate of trust and confidentiality, which in turn enabled spontaneous, open, and deeply engaged participation by the interviewees.

The interviews were semi-structured, conducted with the help of an interview track divided into three sections: before, during and after internment. The procedure and interview template were approved by the Ethics Committee of the Department of Psychology of Developmental and Socialization Processes at Sapienza University of Rome. The interviews were conducted in the period from 1 January 2021 to 30 June 2022.

In order to investigate the social dimension of space and time management in the internees' daily life, the interview analysis adopted a hermeneutic approach, focusing on three specific areas: the structure of the camp and barracks; the scansion of daily rhythms and work activities; the process of attachment to the place and its role in the construction of individual and collective remembrance and personal and social identity.

Although this study does not adhere strictly to ethnographic methods, it draws on ethnographic sensibilities, particularly in its attention to the affective and embodied dimensions of memory as they emerge in retrospective narratives (Goffman, 1961). The objective is not to reconstruct lived experiences in a realist sense, but to remain attuned to the ways in which survivors re-live and re-configure those experiences through the act of narration.

The interviews are not approached as mere recollections of past events, but as emotionally charged narratives through which individuals access and express the cultural and affective frameworks that give value to their past (Pugh, 2013). These retrospective accounts are inevitably shaped by present identity concerns, by the demands of dignity and recognition, and by the emotional labor of revisiting traumatic memories—particularly in contexts where silence or suppression has prevailed. Yet, even if refracted through present-day lenses, such narratives offer a rich window into the processes through which people reconstruct and reinterpret their social worlds in relation to memory, justice, and identity.

The interview setting is thus treated as a relational and performative space in which affect is enacted as much as narrated. Emotions are not considered secondary expressions but are understood as embedded in discursive patterns, bodily gestures, hesitations, silences, and the expressive quality of language itself (Wetherell, 2012). These elements are read not as ancillary to meaning, but as constitutive of the narrative form and content, shaping how trauma is verbalized, re-worked, or resisted.

The analysis is grounded in an interpretive sensibility that listens closely not only to the content of speech, but also to its tone, rhythm, and cadence. Particular attention is paid to how emotional textures emerge through voice and posture, especially in accounts of suffering and resilience (Ellis, 2004; Frank, 2010). Emotions are treated not as private internal states passively reported, but as socially mediated and culturally patterned practices that position the speaker in relation to their past and to their audience.

This approach, however, carries with it important epistemological considerations. Memory is recognized as fluid and relational, continually shaped by the subject's present self-understanding, by the immediate context of the interview—including the presence of the researcher, mediators, or psychological professionals—and by the broader discursive field that renders certain forms of remembering more speakable than others (Portelli, 1997; Riessman, 2008). Narratives are therefore not taken as direct records of past facts but as dialogically produced accounts, co-constructed in the interview encounter.

Nevertheless, it is precisely through this emotionally textured and intersubjectively shaped reanimation of the past that deeper sociological insights can emerge into the enduring effects of violence and internment. Rather than dismissing affect as methodological noise, this approach foregrounds emotion as a key analytical lens for understanding the interplay between individual memory, collective narrative, and political silence.

With regard to the technical dimension of the analysis, the interview transcripts were first translated from Albanian to English. An initial phase of open coding followed, which was subsequently reviewed and refined through thematic analysis by an experienced researcher.

Before coding began, each member of the research team became familiar with the data by independently reading all the interview transcripts. Subsequently, the transcripts were analyzed inductively to identify those perceptions and experiences that were most significant and recurrent (Maguire and Delahunt, 2017).

Analytical induction, an inferential approach to identify relevant issues and develop appropriate conceptual categories to describe and understand the phenomenon under study, was used to carry out this identification (Johnson, 1998; Katz, 2001). This type of inference is characterized by forming an inductive hypothesis to define the conceptual category. Initially, this hypothesis is formulated through the analysis of the empirical material collected. Its appropriateness is gradually specified during the comparison of all the cases observed, identifying some of the common characteristics considered useful for understanding the phenomenon being defined (Acocella et al., 2016).

Through the analytical induction approach, initial codes were generated by dividing the transcribed texts into meaning units^2^. Subsequently, the meaning units were summarized into themes, refined and finalized into descriptive themes.

Additionally, a form was completed for each respondent in order to record specific information relevant for interpretation and analysis. This form contained a concise abstract of the content of the interview, as well as the following information: gender; age; level of education; years of education within the camp; years of education after leaving the camp; period of time he/she lived in the camp; name of the camp he/she lived; how many years he/she lived in the camp; the reason of being part of the camp; typical day in the camp from morning till evening.

In the report, to ensure the anonymity of the interviewees, the interviews were coded with a sequence number (e.g., interview 2), the sex (M/F) and the age of the interviewee (e.g., 60).

Dialectal or idiomatic expressions recur in interviews and can hardly be translated literally. For this reason, when encountering these types of expressions a brief explanation will be given in brackets and italics to distinguish it from the interviewee's quotation.

## Confinement and living conditions of internees

The interaction with physical space plays a crucial role in self-construction. According to the “full ecology” (Bonnes et al., 2009, p. 3), the place an individual resides in not only shapes their relationship with nature and the environment but also delineates the boundaries and possibilities of their behavior. This, in turn, contributes to the social and emotional dimensions of daily life, serving as a fundamental reference in identity formation.

Forced confinement within a specific place, while initially disrupting established norms, profoundly impacts self-construction, leading to intense alienation. This confinement not only results in exclusion from civil life but also triggers a profound alteration in the reciprocal interactions between the individual organism and the social and physical contexts, crucial components in defining personal identity.

The consequences of imprisonment within the internment camps of Enver Hoxha's communist Albania should be read in this light. They were instruments of the dictatorship to conduct a violent campaign of control and repression of dissent. In the camps lived those who were deemed by the regime to be “enemies of the people”, who were treated with “class differentiation” and dehumanization, also implemented through the construction of an extreme living environment.

In his study of Nazi concentration camps, Bettelheim (1943, p. 417) identifies several distinctive features of the extreme conditions experienced by camp prisoners, including deliberate torture, malnutrition, forced labor, comprehensive control over their lives, and lack of access to adequate medical care (Hodgkins and Douglass, 1984). These same peculiarities characterized the lives of prisoners in the internment camps of communist Albania. However, unlike the Nazi concentration camps, the Albanian internment camps are distinguished by their unprecedented duration of more than 40 years.

Between 1945 and 1992, there were about a 100 internment camps throughout Albania. The most famous were the camps of Kruja, Berat, Tepelena, Kamza/Valias, Porto Palermo and Savër. This last one on which the study focuses, unlike other internment camps, was located close to a population center, the municipality of Lushnjë, situated in the south-central part of Albania, in the Fier region.

The interviewees describe the Savër camp near Lushnjë as a village surrounded by barbed wire and bordered by a large moat filled with water. The area was divided into two quarters, the block of internees and the block of freedmen whose task was to control them:

≪I said that Savër was divided into two factions, so to speak. The main street was the barracks of the internees, ballists or reactionaries as they were called, with all sorts of epithets, and the freedmen, those who had come to guard or guide us who were interned≫ (interview 13_F/70).

This camp structuring reflects one of the essential characteristics of the typical environment of total institutions: the strict distinction between the social life of the staff and that of the internees. Both shared the same places, but under radically different conditions. While the freedmen lived in stone-built houses, the internees lived in real barracks.

≪Savër was a camp divided in two. That is, there was a quarter built on barracks and a quarter built on stone houses. The barrack quarter was called the internees' quarter and was a dense group of seven barracks where each barrack had about 16–17 families. In Savër, which according to my idea had up to 1200 inhabitants, more than 800 were interned≫ (interview 26_M/65).

The barracks were in a very precarious condition, built of wood and straw or, later, asbestos cement, and often did not have a proper roof. Thus, people were highly exposed to climatic adversities: the severe cold during the winter and the oppressive heat of summer. In addition, the section of the internees had many inhabitants and was heavily overcrowded: numerous families shared the same barrack and at least 10 people resided in each small room. As reported by one of the interviewees, who recounted the experience with a measured yet emotionally weighted tone, marked by pauses and subtle vocal hesitations, the inmates performed all domestic activities within the same room and were continuously supervised. Toilets were shared and there were not enough toilets for the number of people using them:

≪The apartment was... misery. We were all in a 3x3 room with my father, mother and us kids. In summer with the heat and in winter with the cold. When you cooked there, the smell of paraffin came from those furnaces that were there then. All night long you could smell the paraffin because it had to be burnt to cook. And if you wanted to go to the bathroom, there would be four bathrooms for 16 families≫ (interview 14_M/71).

The furniture and utensils in the barracks were makeshift: there were no real beds, only wooden planks, and straw beds; the floor was made of compacted earth and every object or piece of clothing was placed on precarious planks, filled with mud or red earth. In addition, everyone was deprived of personal belongings before entering the camp.

There was no freedom of movement even within the village itself. Movement was only permitted to go to work in the fields and to attend appeals that were held in the morning and evening. In the evening, movement was restricted only to go to the communal toilets and inside the respective barracks.

The internees were constantly malnourished and did not have access to adequate medical care, which was not a secondary aspect given the overcrowded living quarters, the scarcity of hygienic services and an inadequate diet in relation to the heavy work they were forced to do. The survivors' testimonies describe the restrictions they suffered on a daily basis, including difficulties in accessing medical care in case of health problems:

≪Health problems remained hostage in my heart *(the interviewee intends to express how health issues have been a source of great suffering for him, leaving an indelible mark, an invisible scar)*, even if you were sick, you had to ask permission from the brigadier, the brigadier would give permission, you had to go to work when you were sick because it was a necessity of life. Even if they gave you permission to go, they didn't report that it was necessary to stay for 2–3 days' rest, they wouldn't give it to you because you were the enemy, it was like wearing a wolfskin *(by this expression, the interviewee intends to emphasize how the society outside the internment camp saw the internees as malign and identified them with the enemy)*≫ (interview 3_M/65).

While recounting this harrowing experience, the interviewee lowered his eyes and paused several times, visibly struggling to maintain composure. His hands remained clasped tightly on his knees, a gesture that conveyed emotional containment and unresolved anguish. His voice trembled at times, especially when recalling the expression “wolfskin,” revealing a deeply embodied sense of exclusion.

This excerpt unveils a typical mechanism of the total institution, the infantilisation of the internees who, being in a position of total dependence, had to ask permission to perform any action. This total subjugation of the body and will exemplifies what Foucault (1975) defines as the disciplinary mechanisms of power that operate through surveillance, repetition and constraint, producing a docile and objectified self. In addition, survivors recount that between a request and a response the time was always stretched, because each small question was submitted to different hierarchical ranks and often the response did not arrive in time with respect to what was needed. Maintaining this state of subjection was functional in crystallizing the map of power and emphasizing the difference in status between internees and freedmen. Several interviewees also report a sense of uncertainty that always remained with them. This did not only relate to the precarious living conditions, exposure to the weather, sacrifices and illnesses, but above all to the inability to control anything in their lives, including basic physiological functions. Systems of physical humiliation were systematically implemented. The most common involved the rationing of food, but also the prohibition to go to the bathroom when needed, especially during work.

≪What I remember is that I was always hungry. There was very few food (...) The greatest suffering was when they took us into the woods to work, a long, hard job in which we could not go to the toilet when we wanted or even eat≫ (interview 4_F/74).

As she recounted this, the emotional weight of the memory became visible: she let out a long sigh and slowly shook her head, as if trying to dislodge the painful recollection. Her voice softened and her shoulders curled inward in a protective gesture, while the rhythm of her speech grew irregular, punctuated by brief silences that conveyed distress more powerfully than words alone.

Moreover, the food supplied was not only insufficient but of such poor quality that even the freedmen were outraged. This outrage is exemplified by the reaction of a doctor working at the camp, who upon witnessing the unhygienic conditions of the internees' food, acted with boldness and indignation:

≪One day a doctor came (...), he was a very good doctor. He came to that big courtyard where the food was being prepared and found that big cauldron of bulgur (like couscous) and worms, he saw it and called the officer and the cook and said: “Either keep these people as people, or shoot them”, then he bravely kicked that cauldron and overturned the red wormy mash≫ (interview 4_F/74).

While recounting this scene, the interviewee briefly smiled, but it was a bitter smile, not of amusement but of irony mixed with relief. Her hand gesture mimicked the doctor's kick, indicating that the memory of that moment had been replayed many times in her mind. The gesture had a performative quality, suggesting that this was one of the few instances in which she had witnessed an act of dignity being defended.

Moments like this, rare and symbolically charged, stand in sharp contrast to the overall reality described in the interviews. A stark depiction of life within the internment camps, characterized by the complete violation of human rights, emerges from the analysis of the interviews. Imprisonment was in fact systematically used for the self-spoliation and alienation from civil life: the forced confinement and uprooting from one's original environment destabilized the individual's sense of identity, inducing a profound psychological rupture that severed the individual's ties to the place that once provided continuity and emotional security. According to Fullilove (1996), this process of uprooting is deeply traumatic, stripping individuals of the geographical and social anchors that constitute their sense of self, thereby destabilizing their very existence within the world. In the internment camps of Albania, this radical disconnection from familiar places intensified the alienation of the internees, who were subjected to overwhelming feelings of loss and disorientation. Forced to leave their homes and uprooted from their living contexts, internees were deported to camps where they lived in dilapidated barracks. Moreover, the total control exerted over every aspect of their lives radically suppressed autonomy in space management and drastically restricted freedom of movement. The overcrowded, unsanitary living conditions, coupled with malnutrition and lack of adequate medical care, contributed to their mortification, with severe physical and psychological repercussions.

However, as the interviews report, those who survived often gradually became accustomed to these harsh living conditions after an initial period of shock. This progressive erosion of dignity and habituation to inhumane living conditions is clearly reflected in the following interview excerpt, where the interviewee's tone combines fatigue with quiet resilience. The pauses in his speech convey both the heaviness of memory and the conscious effort to maintain composure while recounting painful experiences. This paralinguistic aspect enriches the narrative, unveiling the emotional complexity underlying the adaptation to extreme hardship.

≪Honestly, when I think back, I don't know how I survived. We didn't have a roof, we didn't have enough food and we couldn't move. In the beginning it was so hard. I was crying, we were crying. I was no longer myself. But then we gained strength. Maybe with time we got used to it. I don't know. Some died, but I don't know how now, but we adapted and survived. We went on together. We felt united≫ (interview 21_M/74).

On the one hand, the process of adaptation consisted in modeling oneself and one's actions according to institutional dictates and observing the restrictions imposed. On the other hand, adaptation consisted in seeking alternative strategies of resistance. The bonds of friendship and mutual support established between the inhabitants of the internment camps are part of these strategies.

≪Exiled people like us were wonderful people. They were people who loved us and we loved them, they helped us and we helped them, we cried like a real family. Good and bad, we faced everything. Maybe that also helped us, kept us alive. Love, respect≫ (interview 13_F70).

In this context, love served as a critical existential anchor, offering a source of meaning and emotional strength in the face of extreme adversity. As Frankl and Sipos (1967) emphasized, love can be a powerful force for resilience, capable of providing individuals with a sense of purpose even in the most dehumanizing environments, such as total institutions. In these settings, where life is often reduced to mere survival, love transcended the physical and psychological hardships, offering a vital emotional connection. It allowed the internees to maintain a sense of unity and solidarity, fostering relationships that provided them with emotional sustenance and a means of coping with the overwhelming isolation and alienation they experienced. The bonds formed through love not only counteracted the brutalizing conditions of the camps but also created a space for shared human dignity, essential for their psychological survival.

## Time and everyday life of internees

Another strategy implemented by total institutions for the self-spoliation consists in acting on time (Scott, 2011). The scanning of time constitutes an essential element in the construction of both individual and social consciousness.

The mechanisms of manipulation at work in the Albanian internment camps were very pervasive in this regard. The first consisted in the colonization of everyday life through forced labor and the minimization of free time. The so-called enemies of the people were in fact forced to perform the heaviest jobs without adequate remuneration because they received a lower wage than the rest of the population:

≪We did the hardest work because we were persecuted. The salary was lower for us≫ (interview 6_F/66).

The tone of the speaker is strikingly flat, almost devoid of emotion, suggesting a deep internalization of injustice, where resignation has replaced protest. This emotional numbness seems to mirror the harsh reality of camp life, where the internees were employed in the most strenuous jobs, mainly in agriculture and swamp drainage, and were forbidden to engage in other types of work:

≪In'79 I started my normal life as an ordinary worker and I had no right to do any kind of work other than agricultural work, I had no right to be a professional, not even as a bricklayer, not even as a steel worker, not even as a sheep herder. (...) So, the work was onerous (...), it was the hardest work you do, that is after mining. After prisons and mines, working in agriculture was the hardest work that was done in the Albanian reality≫ (interview 26_M/65).

Another mechanism through which the self-spoliation was implemented was the loss of all temporal markers. The cycles of individuals' daily activities, from eating to sleeping, from working to relaxing, were carried out with an always identical scansion and, in most cases, collectively. People lived without dates, with days that were all the same and without full awareness of the flow of the calendar. The internees had no possibility of stopping work on Sundays or celebrating holidays; they were forced to work continuously throughout the year with rigid and very long hours. In addition, the internees had to perform the jobs assigned to them in all weather conditions with inadequate equipment:

≪In the summer there was that slogan we all knew “Errë e Sabah”. That is, when the sun came up you had to be at work and when the sun went down you had to go home. And many times, the work was too much, too heavy. When we worked in the winter, the boots had holes in them because the material was weak. When you wore it for a fortnight, it got holes in it, they put a cap on it, and it got holes in another place. Imagine working 1 day in November or December in the mud and it all gets into your boots. And cold, cold≫ (interview 14_M/71).

The final repetition “cold, cold” is delivered with a fading voice and a slight pause, as if evoking a physical memory that resurfaces in the body as much as in speech, reinforcing the embodied suffering of forced labor.

The flow of the hours was scanned only by the three rollcalls to which the internees were forced to report at three different times of the day: morning, afternoon, and evening. This manipulation of time, including extremely long working hours, the absence of weekly rest days, and the homogenization of time into undifferentiated days without dates or clear awareness of the calendar's flow, can be understood within the framework of Goffman's concept of temporal regulation as a tool of self-spoliation (Goffman, 1961). By controlling the daily routines and erasing temporal markers, the institution not only imposed a structure of obedience but also disrupted the natural relationship between the individual and their environment, thus preventing the development of a coherent personal and collective identity. In such a context, the rigid repetition of time-based routines prevented internees from experiencing time as a space for individual expression or reflection. The inmates were woken up at five o'clock in the morning to report for roll call in front of the guards, who assigned work tasks to them. In this sense, time was reduced to orders and rules, destroying the sense of a continuous connection between events, and a continuous relationship between past and future. As the interviews reveal, the internees were controlled in every aspect of their lives. When they left the internment camp to go to work or, in the case of health problems, to a medical examination, they had to make sure they returned at certain times. In fact, to prevent them from escaping they had to report for roll call several times a day:

≪I remember that my father had to go to rollcall three times a day in the mornings when there were difficulties (health problems), while the other days he went in the morning and in the evening≫ (interview 14_M/71).

The interviewer recalls this with a muted, even tone, as though describing an ordinary fact of life, revealing how deeply control had been normalized. This routinised intrusion into daily life was further intensified by the extreme living conditions.

The overcrowding of the Savër camp not only eliminated any possibility of personal space, but also denied the right to solitude, even during essential hygienic activities. Every phase of daily activity was conducted in other people's company. This on the one hand ensured massification, on the other hand reinforced the manipulation typical of total institutions, meaning continuous surveillance and being constantly under scrutiny by others chosen by the institution.

Consequently, the life of the internees was characterized by inhuman living conditions and extraordinarily grueling work schedules. However, forms of resistance to this spoliation of time also emerged in the interviews. Evidence of this can be found in the accounts of certain moments within the camp dedicated to the social dimension, to the building of bonds of friendship, solidarity, and even recreational activities. These experiences are often imbued by the interviewees with a semblance of normality.

≪As children we played a lot of hide-and-seek, with seven tiles (*it is a board game*), etc. Then, when we grew up, we focused on reading novels. From an early age, I had a library card in Lushnjë and did not stop reading those Russian fairy tales until I was 12 years old. After this age we started reading novels, romances that were given to us by Savër's sons who were constantly trying to civilise us≫ (interview 4_F/74).

The interviewee's tone lifts here, animated by the memory of reading; the shift in vocal rhythm marks a sudden access to a world of meaning beyond confinement. Another testimony further confirms the existence of daily forms of resistance, in which children's games are an integral part. Indeed, in the following interview excerpt, the speaker moves through these memories with a lighter rhythm, a sign of regained dignity and self-determination within collective play.

≪We were active people and tried to build our social life in our own way. We had created a football field, we played football for our own personal pleasure. Often boys from the camp dreamed of joining the Lushnjë team, but they could not be accepted because they were exiled. There were many boys who were talented and knew how to play. In the little free time, we had either we played chess, or we played backgammon, or we played cards≫ (interview 26_M/65).

Play is an essential part of a child's life experience, as it promotes creativity, social interaction and mental development. Even in Nazi concentration camps, Jewish children sought solace in games. For the children in the Albanian camps, it was the same: as they often had few personal effects, toys assumed a special value, and so did books. Games and books helped to restore a semblance of normal childhood to young people living in abnormal circumstances.

A change in time management occurred in the last decade. Starting in the 1980s, in fact, the regime allowed a slight relaxation of some rules. Thus, among the concessions came the possibility of seeing, even if rarely, a movie. The movie represented a great social occasion, not only because it gave a different view of the world, but because it became the subject of lengthy discussions and new information. In addition, one could listen to the radio, thus also opening knowledge in other areas, such as sport, music and even the Sanremo Festival (a famous Italian musical festival).

≪We secretly listened to the Sanremo Festival, listened to Italian songs and we performed them together. We got together as a group and celebrated New Year's Eve in each house. So, even in those conditions we tried to enjoy life more or less≫ (interview 4_F/74).

≪For example, in'79-'80 my father was offering an Italian course to a good number of boys there. (...) learning a foreign language became a goal (for the internees), we spread it. There were people engaged in creativity and sometimes we even talked, discussed sports, music, literature. That is. There was no system of values protected in any way through clubs or, so to speak, established organizations. But so, individually, or socially, groups were created for everything. Films were discussed, books were discussed, there were evenings. For example, we used to meet in a corner. We had a corner where, for example, every evening we would discuss a film and express all our thoughts. Or someone, for example, older would come and explain to us that he had seen, how he had seen it, how he had transmitted it. (...) So, social life in a dictatorship is a bit extreme. However, and what little social life there was, it was, so to speak, a little vulgar, a little... but there were people who loved that kind of life and it was, as it were, a spoonful of sugar (*this is an idiomatic phrase that could be translated as “a breath of fresh air”*)≫ (interview 26_M/65).

The tone in which this memory is pronounced becomes layered: halting, nostalgic, ironic. The frequent pauses suggest careful reflection, while the final expression is delivered with a faint smile in the voice, hinting at a bittersweet affection for those stolen moments.

Despite their condition of extreme deprivation and great physical and mental suffering, the internees put in place forms of resistance against time nullification. Although limited by the overarching control of the total institution, these forms reflect the capacity of individuals to reclaim agency in situations of extreme oppression. They are evidently secondary adaptation mechanisms (Goffman, 1961, p. 119), where individuals assert control over certain aspects of their lives, such as time, despite the constraints imposed on them. In the case of internees this involved carving out some time for themselves and asserting small shreds of autonomy, thereby resisting the totality of institutional control. By doing so, they could affirm a sense of identity that stood in contrast to the homogenizing demands of the institution were able to assert a differentiated identity side with respect to institutionalized dictates, and cultivate, within the limits of deprivation situations, some personal interests and affections. Goffman's (1961) framework emphasizes how such adjustments, though subtle, become vital forms of survival and resistance, allowing persons to assert their individuality even under oppression through everyday practices.

## Identity and relationship to place

≪Savër is a dear place to me, despite what I went through, I spent my youth there and I still live there today. I am grateful to the people of Savër for everything they have done for us≫ (interview 5_M/72).

This sentence, expressed by one of the survivors in a subdued yet determined manner, may seem paradoxical. The place that caused the greatest trauma in life is also a cherished place that evokes positive emotions. The close connection with the internment camp is due to a peculiarity of Albanian oppression: compared to other extreme places created by the most diverse totalitarianisms, the camps we are talking about existed for a very long time: a period of more than 40 years. Under the dictatorship of Enver Hoxha, entire generations were born and grew up in these places.

It is precisely the extended duration of this experience that may partly explain the sense of attachment reported by the interviewee and testified to by other survivors. Spending most of their years or, in many cases, their entire lives in internment camps, it is indeed not uncommon for former internees to have developed a feeling of belonging to the place of internment. Moreover, “Where a person lives in a particular locale over an extended period, that person will often develop feelings of affection for, and a sense of belonging, or being of that place, so that place becomes ‘one anchor of his or her identity' ” (Morgan, 2010: 12; Hay, 1998). This link is therefore certainly activated by significant experiences, such as important events or personal growth (Manzo, 2005).

This attachment paradoxically can be understood in light of the concept of place attachment (Scannell and Gifford, 2010; Hay, 1998), which recognizes how even places marked by traumatic experiences can assume an emotional value when they are rooted in profound existential and relational experiences. In the case of Savër, the long duration of forced residency and the building of strong community ties transformed the camp's space into an “identity anchor” (Morgan, 2010), where the trauma was processed through a collective pride. The recurring reference to Savër as “the place of the heart” reflects a form of social and psychological adaptation that allowed the former internees to maintain a sense of dignity and belonging despite the dehumanizing intentions of the total institution.

This emotional attachment is often conveyed not only through words but also through subtle vocal inflections, moments of hesitation, and changes in pace, which reveal the depth of feeling and the complex layers of memory tied to the place. As illustrated in the following excerpt, one interviewee recounts his attachment with a trembling voice and an intensely emotional tone; his gaze is melancholic yet firm and proud, and his words come slowly, carried by the emotional weight of memories that are as vivid today as when they were first lived. However, the personal biographical aspect is not the only factor that contributes to this sentiment. According to the interviewees, the connection to Savër is also associated with a distinct social dimension: the bond with others and the feeling of belonging to a collective “we”.

≪Now it remains as a place, regardless of the fact that it is the place of exile, it remains in the memory after we have lived a long life and it becomes as if we were local because, after all, we created it≫ (interview 7_M/70).

Indeed, the internment camp evokes not only memories of extreme suffering but also positive emotions, particularly those associated with the friendships and mutual support networks formed among the residents. In extreme deprivation contexts, such as those of the internment camps, solidarity was not only a mechanism for practical survival, but also a form of collective psychological resistance. The mutual aid among internees, as described by the interviewees, not only allowed for the sharing of scarce material resources but also redefined an emotional space of autonomy within a coercive structure. According to Bourdieu (1979), solidarity and the formation of social bonds in deprivation contexts act as a strategy of social capital, which not only facilitates physical survival but becomes crucial for the construction of a resistant collective identity. In this sense, the Savër community was not only a place of suffering but also of the creation of shared values and collective resilience, enabling individuals to resist the psychological pressures of the regime's attempt to erase personal and social identities. The daily life that connects the community members to the place also contributes to their attachment to the environment (Scannell and Gifford, 2010). By producing shared historical experiences, values and symbols, each group that lives on the same territory develops a privileged relationship with the place in which it lives, until it becomes an essential reference of its own identity both on a personal and social level. This is even more true within groups that live extreme experiences. In such communities, the sense of we is certainly amplified by the awareness of having a common destiny (Lewin, 1948). It is precisely the consciousness of such deep sharing that leads some survivors to even develop a sense of pride concerning the place.

≪To tell the truth (...) I feel a sense of pride. When I hear the name Lushnjë, I feel my heart beating with joy. I feel luxuriant more than any other province. The people of Lushnjë are a wonderful people. I feel only respect for the people of Lushnjë. As for Savër, as long as I am alive, there is my childhood, there is my life, there is my dream. That is where all the suffering, all the stress, all the bad things started. Nevertheless, I feel nostalgia for Savër. (...) I feel love for Lushnjë. I feel a respect, I feel a love. When I listen to Lushnjë, my heart beats. Even though there we suffered and saw endless suffering≫ (interview 13_F/70).

The pride of place is the positive emotion experienced toward the physical environment with which an individual identifies. This feeling often develops during childhood and through particular place-related activities, with places having relevant social and religious significance and satisfying the individual's psychological needs (Bonaiuto et al., 2019). It is thus an emotion that is deeply rooted in personal and group identities, associating on the individual level with the development of self-confidence and, on the social level, with the development of social identity. In the case of Savër, even if in an apparently paradoxical way, pride of place intervenes as a social-psychological adaptation of the internees aimed at satisfying basic needs in the extreme conditions of camp life, activating an identification with the community of internees which, through mutual support, allows them to survive forced confinement. This is why, paradoxically, in the interviewees' memories the place of deprivation becomes an experience to be proud of because, thanks to the community bond, it made it possible to resist abuse and even to form a social and place identity that is not completely overridden by the regimentation of the total institution.

In some interviews, this bond with place and community is so intense that it takes on a spiritual meaning. For some, the internment camp can in fact be likened to a religious bond with a sacred place (Mazumdar and Mazumdar, 2004). Even today, former internees return to the places of suffering almost annually to keep the memory alive. They carry out these visits in groups, scheduling appointments from different parts of the world where they reside or making the journey every time they return to Albania, as if it were a genuine pilgrimage. The tone of voice when recalling these visits often softens, tinged with nostalgia and reverence, while pauses and subtle shifts in intonation reveal the deep emotional connection that transcends mere words.

≪The first thing I do whenever I return to Albania is to go back to Savër. That even in Lushnjë I had friends, wonderful guys. In Savër I also find those who were communists, I sit with them and open these conversations and laugh, because one must not forget everything. Always without poisoning yourself inside. Because revenge is the greatest enemy man has inside. (...) Even if you take away that camp written here, Savër, I feel a... I have that camp in my heart forever. Savër has been haunted so much. I have Savër and Lushnjë in my heart and I will have them forever. Visiting Albania and not visiting Savër is impossible, it cannot exist≫ (interview 14_M71).

The attachment to these places, which almost become sacred to the internees, results from both the process of adaptation for survival in extreme conditions of deprivation and physical and mental suffering, and the construction of their own identity. This construction is also facilitated by the essential role of the community to which they belonged. In particular, the construction of portions of their social identity and their identity of place to which the very intense attachment to place that has been revealed can be ascribed: a typical expression of place attachment is in fact the need to return to one's place of attachment.

## Conclusions

The research on Savër internment camps in Lushnjë during Enver Hoxha's communist dictatorship (1944–85) reveals resistance to total institutions, including time manipulation, standardization, and spatial exclusion, showcasing manifestations of subjectivation and opposition to self-spoliation.

Goffman (1961) asserts that space and time are pivotal tools within detention organizations, where regimes degrade personal and social identity, deeply affecting each individual. This study confirms how, as noted by Moran (2013) and Haney (2018), the reconfiguration of space and time in carceral settings generates a dislocation of self-perception. The internment camp, therefore, does not simply impose physical containment, but becomes a device for identity erosion, operating through emotional deprivation and symbolic marginalization. Upon entry, recruits undergo a series of desecrations involving the stripping of self, severing ties with the outside world, and the imposition of a standardized identity, lacking freedom in organizing lifetimes and interpersonal relationships. Survivors of the Savër camp accurately reported these mechanisms, including physical confinement, severe movement restrictions, regimented lifetimes, cancellation of time markers, overcrowding, and standardized needs—systematic tools manipulating and erasing personal and social identity in favor of institutionalized reprogramming. As Crewe (2011) emphasizes, spatial regulation in such contexts has profound psychosocial consequences: the denial of privacy and autonomy leads to emotional humiliation and helplessness. This emerges clearly from the testimonies in Savër, where the impossibility of inhabiting space personally deeply undermined individual agency and self-definition.

However, the study also showed the other side of the coin: in the Albanian communist internment camps, people did not only have passive leveling, but—acknowledged in more recent literature that applies Goffman's framework (Elliott, 2012; Scott, 2011; Shreeya, 2018; Smith, 2006)—they also activated certain agentic processes of alternative identity construction.

In particular, the research on Savër highlighted the centrality of communitarian bonds. The interpersonal links established between the inhabitants of the internment camps can in fact be recognized as real strategies of resistance to institutional dictates. There are two mechanisms through which these links have enabled survival.

The first is mutual support. From a Darwinian perspective, it is fundamental for self-preservation. In extreme contexts, the ability to create social relationships is a factor that can increase the chances of survival (Bělín et al., 2022; Davidson, 1980). These survival strategies also respond to the institutional imposition of “dead time” (Jewkes, 2005), which deprives individuals of personal temporal agency. The resistance observed in Savër can be interpreted as a reappropriation of time and identity, opposing what Fiddler (2011) defines as the emotional disempowerment rooted in carceral time discipline. Indeed, studies state that ≪only in rare instances was survival a purely individual achievement. In most cases survival was due to the operation of social factors≫ (Abel, 1951, p. 155). Indeed, for those interviewed, mutual support seems to have had a significant impact on the ability to adapt to the deprivation and extreme living conditions experienced in the Savër camp. For example, people tried to pool what few possessions and talents they had at their disposal: small tools, clothes, shack maintenance work, skills, etc.

The inmates developed a crucial social mechanism for survival through the solidarity that emerged among them. The shared affective life in Savër, however, allowed for the reconstruction of emotional integrity and helped mitigate the fragmentation of identity that carceral logic imposes. This sense of unity and sharing not only helped establish emotional barriers against abuse, providing vital strength for the victims (Davidson, 1980, p. 2), but also nurtured a collective “us identity”. Following the initial trauma of entering the camp system, internees formed new groups and trust bonds, creating alternative social structures functional enough to maintain essential social vitality and a minimum of moral sanity despite challenging conditions (Des Pres, 1980, p. 142). Similar to contexts such as Nazi concentration camps, community coping mechanisms (Klein and Reinharz, 1972) empowered victims to resist the regimentation of living times and space management, activating a new form of group identity resistant to that imposed by the totalizing regime. These are the “secondary adjustments” (Goffman, 1961, p. 189) allowing internees to adapt and make institutional life as comfortable as possible, even in extreme deprivation. These practices manifest in a variety of ways, including secret rituals, symbolic gestures and shared silences. These allow inmates to give new meaning to institutional time and space. As Crewe and Bennett (2012) contend, these “emotional geographies of resistance” proffer potent elements of coherence and individual significance within coercive systems.

A further example of the importance of community in identification processes is place attachment. Unexpectedly, interviewees reported strong feelings of positive attachment to the place of deprivation in which they lived, describing the internment camp almost as a sacred place that contributed to their personal and community growth. Indeed, as the literature indicates, ≪place attachment does not only operate at the level of the individual. Instead, it is also the profoundly social, collective ways of sharing, discussing, and debating memories of place (…) that make (and break) senses of belonging and place attachment. Indeed, it is precisely the intersection of these two elements where bodily subjectivity, embodied knowledge, individual and shared memory come together, that I argue is what makes place attachment so relevant for building a sense of belonging and identity≫ (Degnen, 2016, p. 1647).

In this light, in the interviews, the terrible experience of the ex-internees of the Savër camp is not removed, but gives way to positive feelings of nostalgia, love and even pride toward times and places that are considered fundamental for one's human and social growth.

The shared, painful experiences, both physically and mentally, serve as a unifying factor for all interviewed ex-interns, who view these ordeals as opportunities for human learning. The period of deprivation and suffering transforms into a time where fundamental experiences in human and social relations, solidarity, deep friendships, artistic, cultural, recreational, and carefree moments were lived memories they cherish with love and nostalgia.

Similarly, the places of deprivation, suffering, and humiliation take on a strong positive value, becoming inseparable from one's identity. These locations are not just reminders of survival but integral to personal growth and a sense of belonging to the community. This profound attachment to time and places, for those interned by Hoxha's regime, becomes not only a significant element of memory but also a foundational aspect of their personal and social identity. This phenomenon aligns with the emphasis in positive psychology on the role of traumatic environmental experiences in building resilient individuals (e.g., Lindell, 2012).

Importantly, this study's interpretative approach highlights how memory in this context is far from a passive or static recollection. Instead, it emerges as a dynamic, embodied, and affective process through which internees actively re-signified and resisted the imposed identities and conditions of confinement. Memory here functions as a resource of emotional and identity reconstruction, allowing survivors to reclaim agency and reshape their narratives in the face of trauma and institutional violence. This understanding enriches the classical theoretical frameworks on total institutions by emphasizing the situated and meaning-making nature of memory, deeply intertwined with present social and political contexts as survivors narrate their experiences. Such an approach calls for methodological sensitivity and empathy, recognizing memory's complexity in trauma research and the active role of survivors in narrating their past.

In this perspective, the conceptual value of self-spoliation emerges as central to interpreting the lived experience of internees. More than a descriptive term, self-spoliation captures the systematic dismantling of personal identity operated by total institutions, encompassing the deprivation of autonomy, emotional disconnection, and the dissolution of social roles. Yet, this process also becomes the ground on which subjects resist and reconstruct the self through bonds of solidarity and shared meaning. As such, self-spoliation is not only the sign of annihilation but also the conceptual threshold through which resilience, memory, and re-signification of the self become possible.

The strong awareness of this process of attachment to the time and place of the suffering and deprivation of the ex-internees in the Albanian camps represents one of the most powerful and effective elements in the defeat of the horrors that the Albanian dictatorship, as well as all other dictatorships, could produce.

## Author's note

This paper is the result of joint work by all authors. However, for formal academic attribution, it is specified that Federica Floridi wrote the Introduction, Confinement and Living Conditions of Internees, and Methods; Silvia Cataldi authored Time and Space in Total Institutions and Time and Everyday Life of Internees; Marino Bonaiuto authored Identity and Relationship to Place; Alessandra Talamo authored the Conclusions.

## Data Availability

The datasets presented in this article are not readily available because the data used are interview transcripts that contain a lot of sensitive information that cannot be disclosed. Requests to access the datasets should be directed to Silvia Cataldi, mailto:silvia.cataldi@uniroma1.it.

## Footnotes

^1^ https://iskk.gov.al/

^2^In qualitative content analysis, meaning units are defined as segments of the text that are related by their content and context, and serve as the fundamental building blocks for interpretation. These units can range from a few words to several sentences or paragraphs, depending on their relevance to the research question (Mayring, 2000).
